# Antibodies against vaccine-preventable infections after CAR-T cell therapy for B cell malignancies

**DOI:** 10.1172/jci.insight.146743

**Published:** 2021-06-08

**Authors:** Carla S. Walti, Elizabeth M. Krantz, Joyce Maalouf, Jim Boonyaratanakornkit, Jacob Keane-Candib, Laurel Joncas-Schronce, Terry Stevens-Ayers, Sayan Dasgupta, Justin J. Taylor, Alexandre V. Hirayama, Merav Bar, Rebecca A. Gardner, Andrew J. Cowan, Damian J. Green, Michael J. Boeckh, David G. Maloney, Cameron J. Turtle, Joshua A. Hill

**Affiliations:** 1 Vaccine and Infectious Disease Division, Fred Hutchinson Cancer Research Center, Seattle, Washington, USA.; 2 Department of Medicine, University of Washington, Seattle, Washington, USA.; 3 Clinical Research Division, and; 4 Immunotherapy Integrated Research Center, Fred Hutchinson Cancer Research Center, Seattle, Washington, USA.; 5 Seattle Children’s Hospital, Seattle, Washington, USA.

**Keywords:** Infectious disease, Oncology, Adaptive immunity, Cancer immunotherapy, Immunoglobulins

## Abstract

**BACKGROUND:**

Little is known about pathogen-specific humoral immunity after chimeric antigen receptor–modified T (CAR-T) cell therapy for B cell malignancies.

**METHODS:**

We conducted a prospective cross-sectional study of CD19-targeted or B cell maturation antigen–targeted (BCMA-targeted) CAR-T cell therapy recipients at least 6 months posttreatment and in remission. We measured pathogen-specific IgG against 12 vaccine-preventable infections and the number of viral and bacterial epitopes to which IgG was detected (“epitope hits”) using a serological profiling assay. The primary outcome was the proportion of participants with IgG levels above a threshold correlated with seroprotection for vaccine-preventable infections.

**RESULTS:**

We enrolled 65 children and adults a median of 20 months after CD19- (*n* = 54) or BCMA- (*n* = 11) CAR-T cell therapy. Among 30 adults without IgG replacement therapy (IGRT) in the prior 16 weeks, 27 (90%) had hypogammaglobulinemia. These individuals had seroprotection to a median of 67% (IQR, 59%–73%) of tested infections. Proportions of participants with seroprotection per pathogen were comparable to population-based studies, but most individuals lacked seroprotection to specific pathogens. Compared with CD19-CAR-T cell recipients, BCMA-CAR-T cell recipients were half as likely to have seroprotection (prevalence ratio, 0.47; 95% CI, 0.18–1.25) and had fewer pathogen-specific epitope hits (mean difference, –90 epitope hits; 95% CI, –157 to –22).

**CONCLUSION:**

Seroprotection for vaccine-preventable infections in adult CD19-CAR-T cell recipients was comparable to the general population. BCMA-CAR-T cell recipients had fewer pathogen-specific antibodies. Deficits in both groups support the need for vaccine and immunoglobulin replacement therapy studies.

**FUNDING:**

Swiss National Science Foundation (Early Postdoc Mobility grant P2BSP3_188162), NIH/National Cancer Institute (NIH/NCI) (U01CA247548 and P01CA018029), NIH/NCI Cancer Center Support Grants (P30CA0087-48 and P30CA015704-44), American Society for Transplantation and Cellular Therapy, and Juno Therapeutics/BMS.

## Abstract 2

In this prospective study, we investigated antibodies against vaccine-preventable infections and other pathogen-specific antibodies in individuals with remission after CAR-T cell therapy for B lineage malignancies.

## Introduction

Prolonged deficiencies in humoral immunity are a critical concern in individuals who achieve durable remissions of underlying B cell malignancies after treatment with chimeric antigen receptor–modified T cell (CAR-T cell) therapy (1, 2). Lymphodepleting chemotherapy followed by CAR-T cell infusion is an effective treatment for patients with B cell malignancies. CAR-T cell products targeting the cell surface protein CD19 are commercially available for treatment of relapsed and/or refractory (R/R) B cell non-Hodgkin lymphomas (NHLs) (3–6) and acute lymphoblastic leukemia (ALL) (7). CAR-T cells targeting B cell maturation antigen (BCMA) demonstrate promising results in patients with R/R multiple myeloma (MM) (8).

Individuals who are candidates for CAR-T cell therapy already have a high net state of immunosuppression attributable to their underlying disease and preceding chemoimmunotherapies. CAR-T cells independently contribute to immune deficits through “on-target, off-tumor” effects because of expression of their targets on the surface of nonmalignant cells, resulting in depletion of healthy B cell subsets (9). After CD19-CAR-T cell therapy, CD19^+^ B cell aplasia is nearly universal and may persist for years (4, 7, 10–12). CD19 is highly expressed on naive and memory B cells, whereas its expression is absent or reduced on certain types of plasma cells in the bone marrow, which produce pathogen-specific IgG to previously encountered antigens. Thus, this population of plasma cells may not be depleted by CD19-CAR-T cells and will continue to produce pathogen-specific IgG (9, 13–15). After BCMA-CAR-T cell therapy, there is specific depletion of plasma cells expressing BCMA (8), but BCMA is not expressed on earlier B cell subsets (16). Thus, CD19- versus BCMA-CAR-T cell recipients may have distinct humoral immunodeficiencies.

The long-term implications of sustained CD19^+^ and BCMA^+^ B cell depletion on humoral immunity and infection risk after CAR-T cell therapy are poorly understood. Hypogammaglobulinemia is common after CAR-T cell therapy (10, 12, 17), which has driven frequent administration of IgG replacement therapy (IGRT) (4, 7, 18). However, the utility of IGRT in this context is unclear and may add side effects and expense without benefit (18). Furthermore, IGRT products are a costly and limited resource, underscoring the need for evidence-based utilization (18).

Observations that pathogen-specific IgG levels may not be affected by CD19-CAR-T cell therapy in adults informed our hypothesis that IgG antibodies against vaccine-preventable infections may be preserved after CD19-CAR-T cell therapy (11, 12, 19). In contrast, we hypothesized that pathogen-specific IgG levels may be lower in BCMA-CAR-T cell recipients because of plasma cell depletion by BCMA-CAR-T cells or prior therapies. Understanding the deficits in humoral immunity in CAR-T cell therapy survivors has important implications for their long-term care, including stewardship of IGRT products and vaccination strategies, as underscored by the current SARS-CoV-2 pandemic (20).

In this prospective cross-sectional study, we investigated antibodies against vaccine-preventable infections and other pathogen-specific antibodies in individuals with long-term remission after CAR-T cell therapy for B cell lineage malignancies.

## Results

### Participants and treatment characteristics.

**Figure 1:**
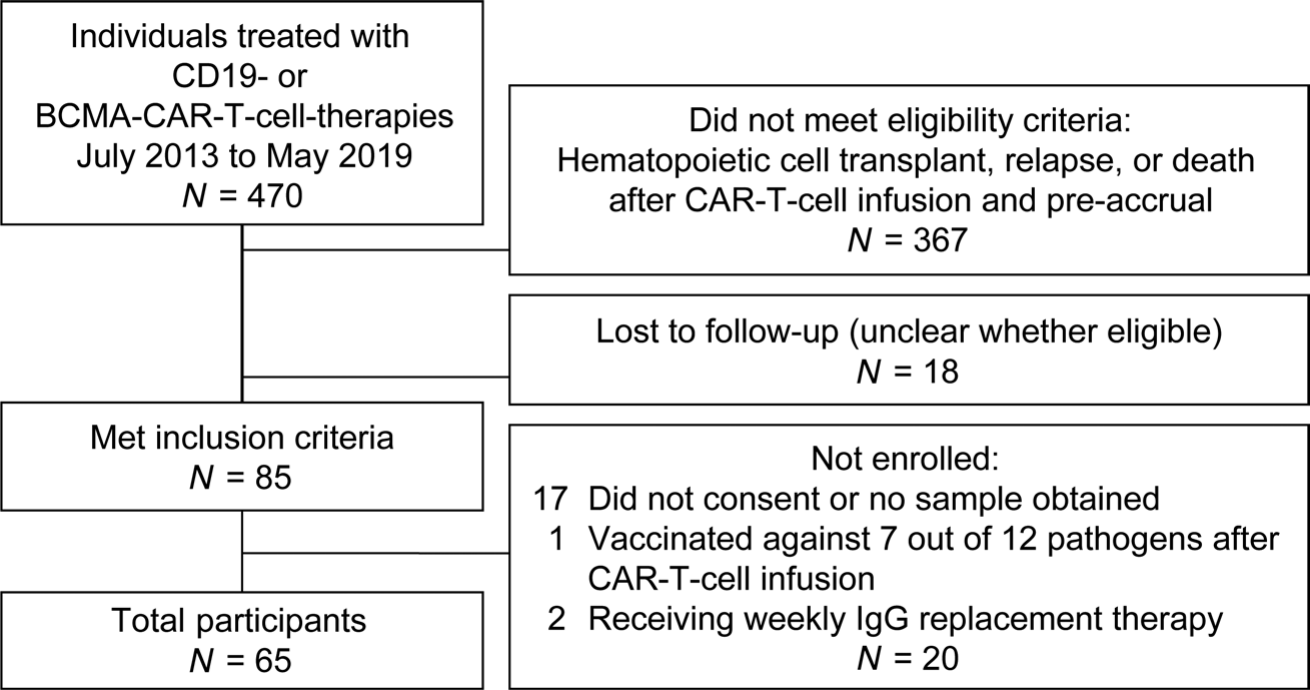
Consort diagram.

Of all children and adults who were treated with CD19- or BCMA-CAR-T cell therapy at Fred Hutchinson Cancer Research Center and Seattle Children’s Hospital between July 2013 and May 2019, 85 were alive, were in ongoing remission, and had not received additional treatments as per our inclusion criteria. We enrolled 65 (76%) of these individuals as indicated in Figure 1. Participants resided in 19 states in the United States and in 3 additional countries. Participant characteristics and treatment protocols are detailed in Table 1 and Supplemental Table 1, respectively. The median age was 59 years (range, 1–76), and 7 individuals (11%) were younger than 18 years old. Participants received a median of 5 (range, 1–21) prior treatment regimens, and 32 participants (49%) previously underwent HCT. The CAR-T cell target was CD19 in 54 participants (83%) with NHL, ALL, or chronic lymphocytic leukemia (CLL) and BCMA in 11 participants (17%) with MM. At time of infusion, 56 of 65 (86%) CAR-T cell products were investigational. Seven of them were later FDA approved (Supplemental Table 1). The median time from CAR-T cell therapy to sample collection was 20 months (range, 7–68). IGRT was administered to 35 of 65 participants (54%) within 16 weeks (≥4 half-lives of IgG) (21) before sample collection and was more frequent in CD19-CAR-T cell recipients younger than 18 years old, BCMA-CAR-T cell recipients, participants with ALL, and participants with a prior allogeneic HCT (Table 1). Among the 30 participants without IGRT in the previous 16 weeks, 16 never received any IGRT and 12 received their last IGRT more than 24 weeks prior (≥6 half-lives of IgG).

### Total immunoglobulin levels.

**Figure 2:**
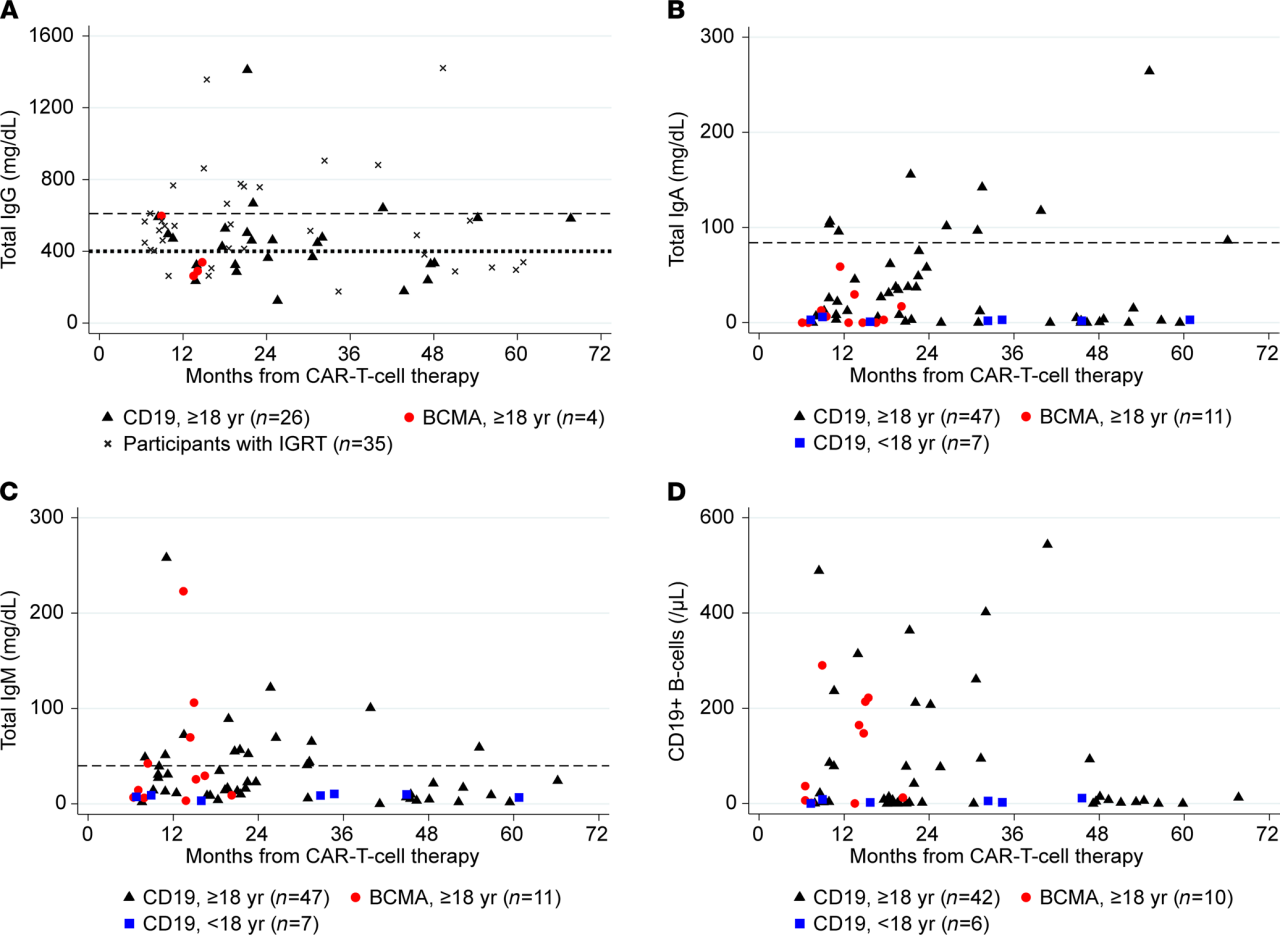
Total serum immunoglobulin levels and peripheral blood CD19^+^ B cell counts. These scatterplots demonstrate (**A**) IgG levels, (**B**) IgA levels, (**C**) IgM levels, and (**D**) CD19^+^ B cell counts based on time after CAR-T cell therapy. Each symbol represents results from a single participant and provides information about CAR-T cell target and age; (**A**) also distinguishes between participants with and without IgG replacement therapy (IGRT) in the prior 16 weeks. The dashed horizontal line at 610 mg/dL illustrates the lower limit of normal (LLON). The dotted horizontal line at 400 mg/dL illustrates the level below which IGRT was recommended per institutional guidelines. Among those without IGRT, total IgG levels were below the LLON in 90% of participants and below 400 mg/dL in 47% of participants, and there was no correlation between total IgG and time after CAR-T cell infusion (Spearman’s *r* = –0.03). (**B** and **C**) The dashed horizontal lines represent the LLON for individuals ≥ 18 years old (84 mg/dL and 40 mg/dL, respectively). There was no significant correlation between serum total IgA or IgM and time after CAR-T cell infusion (Spearman’s *r* = –0.02 and –0.12, respectively). (**D**) CD19^+^ B cell counts among 58 participants with available results. There was no correlation between B cell count and time after CAR-T cell infusion (Spearman’s *r* = –0.11).

Participants who received IGRT closer to sample collection, particularly within the prior 8 weeks, had higher total IgG levels (Supplemental Figure 1). Among 30 adults not receiving IGRT within the previous 16 weeks, IgG was below the lower limit of normal (LLON; 610 mg/dL) in 27 individuals (90%) and below 400 mg/dL in 14 individuals (47%, Figure 2A). Among all 65 participants, total IgA and IgM levels were below the LLON in 55 (85%) and 47 (72%) individuals, respectively (Figure 2, B and C). There were no significant correlations between total IgG, IgA, or IgM and time after CAR-T cell therapy (Figure 2, A–C).

### Peripheral blood B cells and T cells.

Among 58 participants (89%) with PBMC samples, the median absolute CD19^+^ B cell count was 8 cells/μL (IQR, 2–95), and the median percentage of CD19^+^ B cells of peripheral blood white blood cells was 0.09% (range 0%–13.9%). Twenty-three participants (40%) had ≥20 B cells/μL (Figure 2D), consisting of 17 of 42 (40%) CD19-CAR-T cell recipients ≥ 18 years old, 0 of 6 (0%) CD19-CAR-T cell recipients < 18 years old, and 6 of 10 (60%) BCMA-CAR-T cell recipients. Similarly, 19 participants (33%) had ≥1% CD19^+^ B cells of total white blood cells, consisting of 13 of 42 (31%) CD19-CAR-T cell therapy recipients ≥ 18 years old, 0 of 6 CD19-CAR-T cell therapy recipients < 18 years old, and 6 of 10 (60%) BCMA-CAR-T cell therapy recipients. The proportion of individuals with ≥20 CD19^+^ B cells/μL did not vary significantly by proximity of CAR-T cell therapy (Figure 2D).

Among the 23 participants with ≥20 CD19^+^ B cells/μL, the dominant B cell populations were naive (CD27^–^CD38^–^IgD^+^) or transitional (CD27^–^CD38^+^IgM^hi^IgD^lo^) B cells (median, 65% of CD19^+^ B cells; IQR, 58%–76%). Switched memory B cells (CD27^+^IgD^–^) were rare (median, 2% of CD19^+^ B cells; IQR, 1%–3%) regardless of recency of CAR-T cell therapy. These findings were similar in CD19- and BCMA-CAR-T cell recipients (Supplemental Figure 2).

Among these same 58 participants with PBMC samples, 47 (81%) had absolute CD4^+^ T cells greater than or equal to 200 cells/μL. Absolute CD4^+^ and CD8^+^ T cell counts are depicted in Supplemental Figure 3.

### Pathogen-specific B cells.

We also explored the presence of respiratory syncytial virus–specific (RSV-specific) CD19^+^ B cells in PBMCs to understand the recovery of pathogen-specific memory B cells. This virus represents an important pathogen to which most individuals have been exposed. Among the 23 participants with at least 20 CD19^+^ B cells/μL, 15 (65%) had detectable RSV-specific switched memory B cells, consisting of a median of 0.4% (IQR, 0%–1.2%) of all switched memory B cells. Among RSV-specific B cells, the predominant populations were naive/transitional B cells (median proportion, 59% [IQR, 48%–72%]; median absolute cell count, 0.68 cells/μL [IQR, 0.27–2.03]); switched memory B cells were rare (median proportion, 1.3% [IQR, 0%–2.9%]; median absolute cell count, 0.02 cells/μL [IQR, 0–0.04]) and less frequent than in 3 healthy adult controls (range, 7%–18%; Supplemental Figure 2F).

### Antibodies against vaccine-preventable infections.

**Figure 3:**
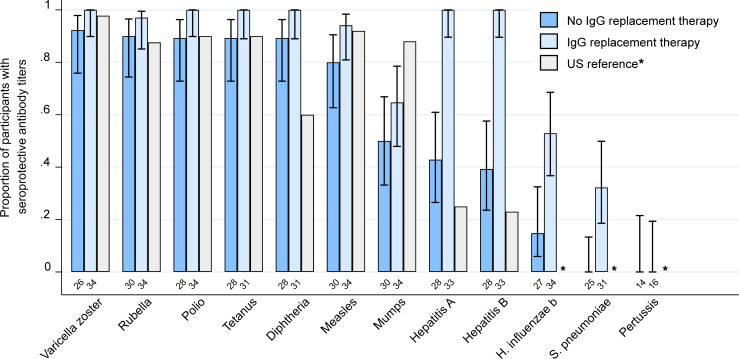
Proportion of CAR-T cell therapy recipients with seroprotective antibody titers against vaccine-preventable infections. Bar graph showing the proportion of participants with seroprotective IgG titers for each vaccine-preventable infection, stratified by receipt of IGRT in the previous 16 weeks. Data from population-based studies in the United States are provided for comparison (22–27). US reference data were not available for *H*. *influenza* b, *S*. *pneumoniae*, and pertussis (indicated as *). Pertussis antibodies were only tested in the first testing batch of 31 participants based on negative results in all samples of the first batch. The total number of participants contributing data to each group are shown below the bars. Whiskers indicate the Wilson 95% confidence interval. Numerical results for participants who did not receive IGRT within the previous 16 weeks are provided in Supplemental Table 5.

**Figure 4:**
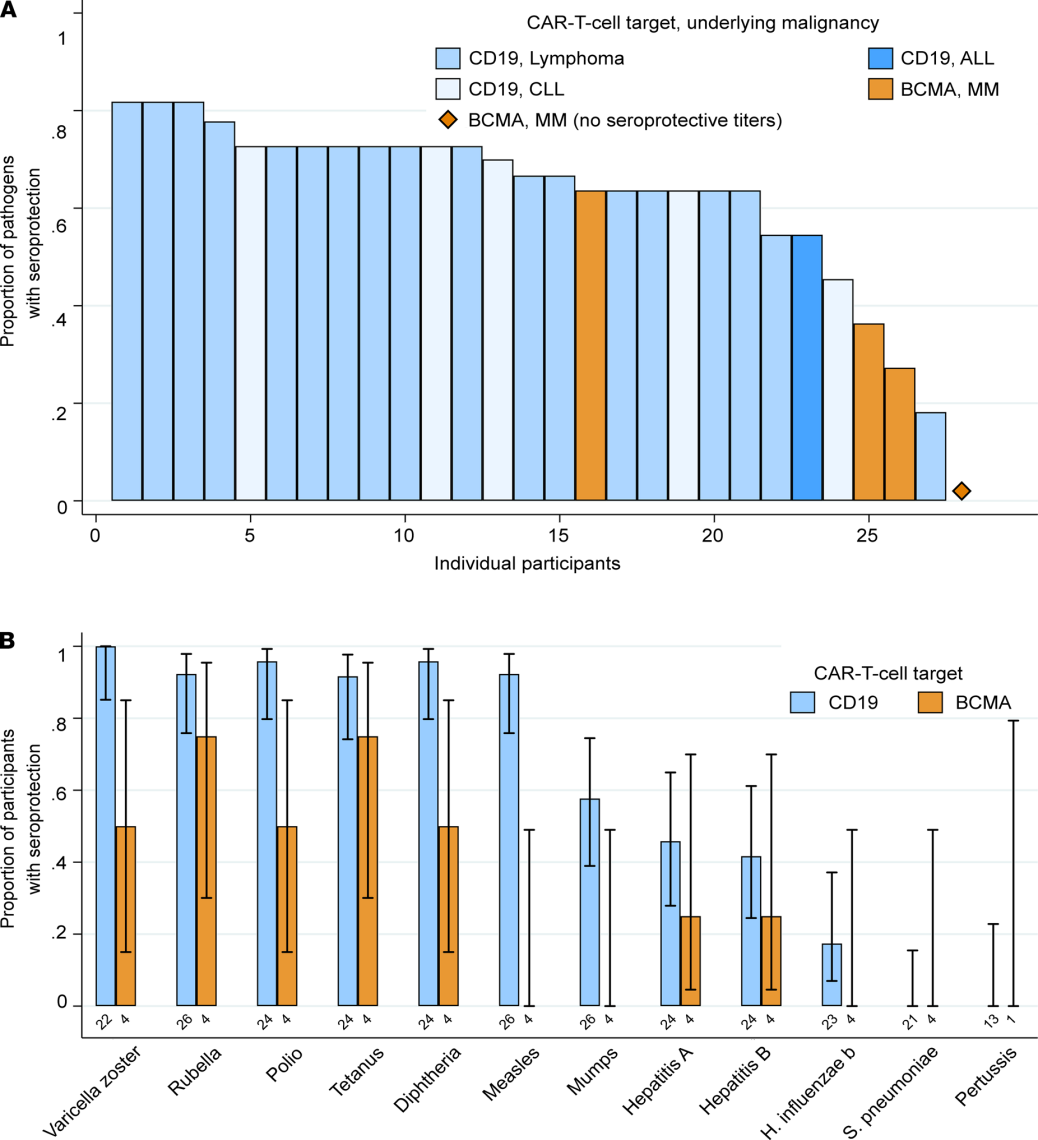
Seroprotective antibody titers stratified by CD19- versus BCMA-CAR-T cell therapy among 30 participants without IGRT in the previous 16 weeks. (**A**) Bar chart showing the proportion of pathogens with seroprotective IgG titers per individual participant. Each bar represents a participant; 28 individuals with at least 6 valid test results are shown. Pertussis results were excluded from this analysis. One BCMA-CAR-T cell therapy recipient had no seroprotective titers. (**B**) Bar graph showing the proportion of participants with seroprotective IgG titers for each vaccine-preventable infection, stratified by CAR-T cell target. The total number of participants contributing data to each group are shown below the bars. Whiskers indicate the 95% confidence interval.

**Figure 5:**
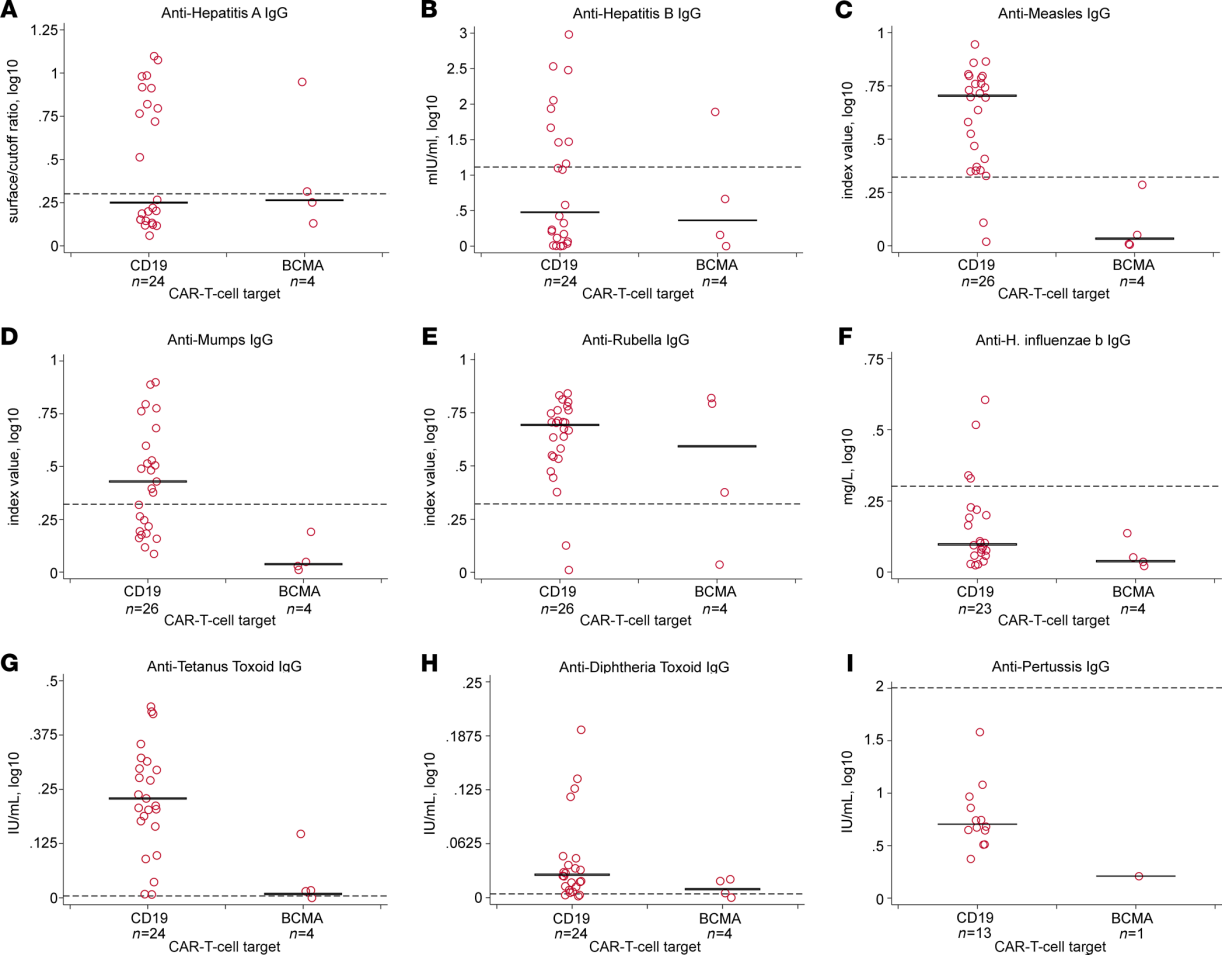
Absolute pathogen-specific IgG titers stratified by CD19- versus BCMA-CAR-T cell therapy among 30 participants without IGRT in the previous 16 weeks. In panels **A**–**I**, solid black horizontal bars represent the median, and horizontal dashed reference lines represent the cutoff value for seroprotection. Each data point represents a participant. Exact numbers of participants providing results per pathogen are shown below the *x* axis labels, and number of participants with seroprotective antibody titers per pathogen is depicted in Supplemental Table 5. Anti-IgG values were transformed using log_10_(value+1). Varicella zoster and polio results are not provided because test results were not quantitative. Results for *S*. *pneumoniae* serotypes are provided in Supplemental Figure 5.

We next tested all participants for IgG against vaccine-preventable infections. Individuals who received IGRT within the previous 16 weeks had a higher prevalence of seroprotective IgG titers, particularly for hepatitis viruses, *Haemophilus influenzae* type b (Hib), and *Streptococcus pneumoniae* (*S*. *pneumoniae*) (Figure 3). Among the 30 adult participants who did not receive IGRT within the preceding 16 weeks, the proportion of participants with seroprotective IgG titers was generally comparable to data from population-based studies in the United States, although some studies used different assays and thresholds to define seroprotection (22–27). Seroprotective IgG titers were detected for a median of 67% (IQR, 59%-73%) of tested pathogens (Figure 4A), despite hypogammaglobulinemia in 27 (90%) of these individuals. The proportion of participants with seroprotection was lowest for mumps (50%; 95% CI, 33%–67%), hepatitis A virus (HAV; 43%; 95% CI, 27%–61%), hepatitis B virus (HBV; 39%; 95% CI, 24%–58%), Hib (15%; 95% CI, 6%–32%), *S*. *pneumoniae* (0%; 95% CI, 0%–13%), and *Bordetella pertussis* (*B*. *pertussis*; 0%; 95% CI, 0%–22%) (Supplemental Table 5). For *S*. *pneumoniae*, seroprotection for each serotype in the pneumococcal conjugate vaccine was similarly low (Supplemental Figure 4). Absolute titer results stratified by CAR-T cell target are depicted in Figure 5 and Supplemental Figure 5. The number of individuals contributing data for each pathogen is detailed in Supplemental Table 5; 41 of 780 (5%) antibody results were excluded because of corresponding vaccination after CAR-T cell therapy and before sample collection.

### Antibodies to viral and bacterial epitopes using a comprehensive serological profiling assay (VirScan).

Among the 30 adult participants who did not receive IGRT within the preceding 16 weeks, the median number of overall “epitope hits” per participant was 259 (IQR, 209–310). The median number of viral epitope hits per participant was 240 (IQR, 190–269) for a median of 69 species (IQR, 54–81). The median number of bacterial epitope hits per participant was 31 (IQR, 23–38) for a median of 11 (IQR, 9–14) species.

### Association of clinical variables with seroprotective antibody titers and epitope hits.

**Figure 6:**
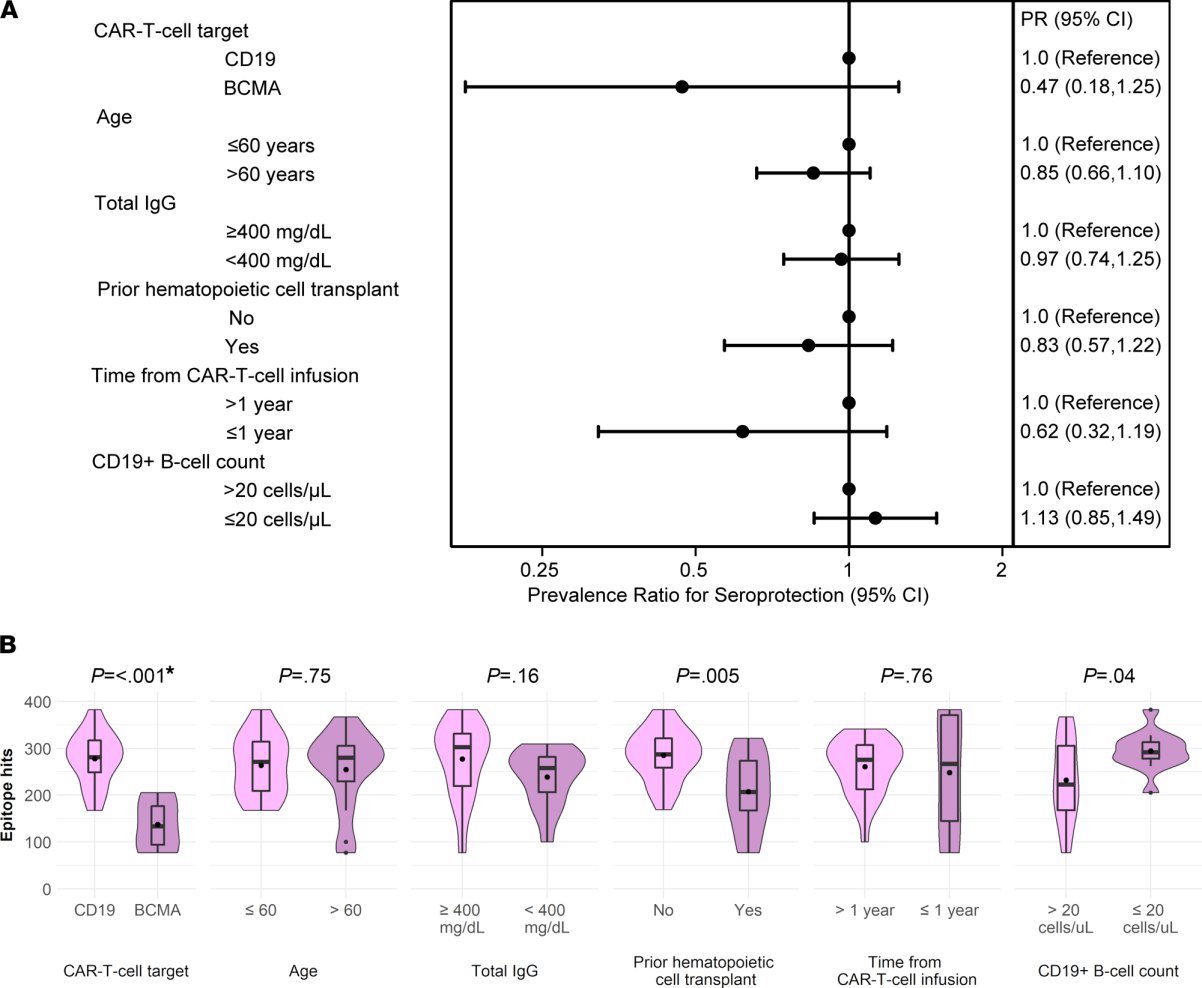
Association of primary clinical variables with seroprotective antibody titers and epitope hits among 30 participants without IGRT in the previous 16 weeks. (**A**) Forest plot demonstrating associations of prespecified variables with prevalence of seroprotective IgG titers to vaccine-preventable infections. Values less than 1 indicate a lower prevalence of seroprotective antibody titers compared with the reference group. For example, BCMA-CAR-T cell therapy recipients had a lower prevalence of seroprotective antibody titers compared with CD19-CAR-T cell therapy recipients, although the difference did not reach statistical significance. Dots represent PR, and whiskers indicate the 95% CI derived from GEE. (**B**) Violin plots comparing the number of viral or bacterial epitopes recognized by IgG (epitope hits) by prespecified variables. Violins show the distribution of the data. Box plots indicate the IQR and median. Dots in the boxes indicate the mean. *P* values are derived from the univariate linear regression model (Supplemental Table 6). Asterisk indicates that the CAR-T cell target remained significant in a linear regression model adjusted for prior HCT, CD19^+^ B cell count, and IgM level (Supplemental Table 6).

To test our hypothesis that pathogen-specific IgG titers may vary by CAR-T cell target, we tested for associations of participant and treatment characteristics with seroprotective titers for vaccine-preventable infections in the 30 adults who did not receive IGRT within the preceding 16 weeks. BCMA-CAR-T cell recipients had fewer seroprotective IgG titers compared with CD19-CAR-T cell recipients, and the only participant with no seroprotective titers received BCMA-CAR-T cells (Figure 4). In a univariable generalized estimating equations (GEE) model, the most notable variables associated with a lower prevalence of seroprotective IgG titers were BCMA-CAR-T cell therapy (prevalence ratio, 0.47; 95% CI, 0.18–1.25) and sample collection within a year of CAR-T cell infusion (prevalence ratio, 0.62; 95% CI, 0.32–1.19; Figure 6A and Supplemental Figure 6A), but these findings did not reach statistical significance. Total IgG level below 400 mg/dL was not associated with seroprotective IgG titers (prevalence ratio, 0.97; 95% CI, 0.74–1.25). In models of additional variables, none were associated with seroprotective IgG titers (Figure 6A). The number of participants per outcome and explanatory variable category precluded adjusted analyses.

These findings were recapitulated in analyses of the number of viral and bacterial epitopes recognized by IgG from the VirScan assay. Differences by primary and secondary variables of interest are depicted as violin plots in Figure 6B and Supplemental Figure 6B, respectively. In an adjusted linear regression model, CAR-T cell target was significantly associated with epitope hits and demonstrated that BCMA-CAR-T cell therapy recipients had fewer epitope hits than CD19-CAR-T cell therapy recipients (mean difference, –90 epitope hits; 95% CI, –157 to –22; Supplemental Table 6).

## Discussion

In this prospective cross-sectional study of individuals with ongoing remission after CAR-T cell therapy for B cell malignancies, we demonstrated that seroprotection for vaccine-preventable infections was comparable to the US population after CD19- but not BCMA-CAR-T cell therapy. The data suggest that BCMA-CAR-T cell recipients had a lower prevalence of seroprotective antibody titers compared with CD19-CAR-T cell recipients, likely due to depletion of antibody-producing plasma cells. In both groups, most individuals lacked seroprotective IgG against a specific subset of pathogens, such as encapsulated bacteria like *S*. *pneumoniae*, that cause substantial morbidity in persons with humoral immunodeficiencies (28). These hypothesis-generating findings identify the need for studies of vaccination and IGRT strategies to determine efficacy and identify patients who may benefit most.

Consistent with our hypotheses, we found that the proportion of adult CD19-CAR-T cell recipients with seroprotective IgG titers to vaccine-preventable infections was comparable to data from surveillance studies in the United States (22–27). Across all participants, we demonstrated a low prevalence of seroprotective antibody titers for mumps, HAV, HBV, Hib, *S*. *pneumoniae*, and *B*. *pertussis*. These findings could be due to lack of prior exposure or vaccination, poor immunologic response because of the underlying disease or prior cancer therapies such as HCT, or rapid waning of immunity (25, 26, 29–34). The prevalence of seroprotective IgG titers was lower in BCMA- compared with CD19-CAR-T cell recipients, but this finding did not reach statistical significance. Although the strength of this conclusion is limited by sample size, we demonstrated a significantly lower number of epitope hits for viruses and bacteria in BCMA- versus CD19-CAR-T cell recipients. There are data demonstrating low pathogen-specific antibody titers in patients with MM who have not undergone HCT, but it is unclear how this compares with other cancer patients receiving chemotherapy (31, 35, 36). In order to better characterize the direct effects of CD19 versus BCMA-CAR-T cell therapy on pathogen-specific antibodies, longitudinal studies designed to account for pathogen-specific IgG titers before and after CAR-T cell therapy and heterogeneity in patient characteristics will be important. Nonetheless, from a pragmatic perspective, this study demonstrates that patients with MM who received BCMA-CAR-T cell therapy have severe antibody deficits.

Our data raise important questions pertaining to supportive care approaches following CAR-T cell therapies based on the CAR-T cell target (37). For instance, vaccination for specific pathogens, such as *S*. *pneumoniae*, may be a sufficient infection prevention strategy in CD19-CAR-T cell therapy recipients. In contrast, IGRT may be more efficacious and cost-effective in BCMA-CAR-T cell therapy recipients. The rationale for vaccination versus IGRT for infection prevention is also dependent on immune reconstitution. As shown in other studies, we demonstrated that CAR-T cell therapy recipients can recover CD19^+^ B cells while in remission (7, 10, 38). Recovery of plasma cells in patients with remission after BCMA-CAR-T cell therapy has been anecdotally observed but is not yet well established. Interestingly, our analyses suggested that on average, individuals may have fewer seroprotective titers if less than 1 year after CAR-T cell therapy compared with more than 1 year (Figure 6A). This could suggest that patients recover immunity over time. However, the comparison was limited by few patients in the early time frame, and results from the VirScan serosurvey did not indicate a difference by time after CAR-T cell therapy (Figure 6B). Among individuals in our study with at least 20 CD19^+^ B cells/μL, the majority of B cells were naive. Switched memory B cells represented only 2% of total CD19^+^ B cells independent of CAR-T cell target, which mirrors proportions seen in young, immunologically immature children (39). This might result in delayed humoral responses to reinfections, indicating the potential need to reestablish a memory B cell pool through vaccination. The same pattern was demonstrated for RSV-specific B cell subsets, and the proportion of RSV-specific switched memory B cells was lower for CAR-T cell recipients compared with healthy controls. Despite that RSV is a pathogen that people are routinely exposed to and to which humoral immunity is expected, 35% of tested participants had no detectable RSV-specific switched memory B cells (40). Evidence of CD19^+^ B cell recovery in almost half of the participants, in addition to absolute CD4^+^ T cells greater than 200 cells/μL in most, support the plausibility of generating vaccine responses. Although CAR-T cell therapy recipients may have weaker vaccine responses compared with healthy individuals, vaccination may nonetheless prevent infections, decrease their severity, avoid hospitalizations, and save lives.

The nature of pathogen-specific immune deficits after CAR-T cell therapies, and the clinical implications, are incompletely understood. The high frequency of prolonged hypogammaglobulinemia after CAR-T cell therapies (10, 12, 17) has driven the clinical practice of prophylactic IGRT in patients with low IgG levels or recurrent infections (1, 2), despite the lack of clear evidence for benefit in secondary immunodeficiencies (41–45). In our cohort of long-term survivors after CAR-T cell therapy, more than half were treated with IGRT within 16 weeks prior to enrollment. However, limited access, potential side effects, and high expense demand careful stewardship of IGRT (18). Our findings build upon prior studies to show that CD19-CAR-T cell therapy may not affect preexisting pathogen-specific IgG production due to sparing of CD19^–^ plasma cells (11, 12, 19). There is evidence that these CD19^–^ long-lived plasma cells can survive for decades independent of the memory B cell pool and are less susceptible to high-dose chemotherapies, HCT, and radiation than other B lineage cells (13–15, 46). Our findings also reinforce the concept that total IgG is a poor correlate for pathogen-specific IgG (11, 19, 47) and challenge the routine need for IGRT based on serum total IgG levels after CD19-CAR-T cell therapy. In contrast, depletion of plasma cells in patients with MM, either from preceding chemoimmunotherapy or subsequent BCMA-CAR-T cell therapy, appears to be associated with substantial loss of pathogen-specific IgG, rendering affected individuals more likely to benefit from IGRT. Children may also have fewer seroprotective antibody titers because of less-established humoral immunity (48), but we were unable to address this question due to concurrent administration of IGRT in all participants younger than 18 years old.

Together, our findings suggest that the risk-benefit ratio of IGRT could be guided by focusing use in adults who both have hypogammaglobulinemia and develop severe bacterial infections or who have poor responses to immune challenge with an exogenous antigen (e.g., vaccination) (18, 36), particularly after BCMA-CAR-T cell therapy and within the first year after CAR-T cell therapy. To date, there are no data pertaining to vaccine responses in this patient population. Based on guidelines for vaccination of immunocompromised hosts including HCT recipients (49–52), and the kinetics of immune reconstitution after CD19-CAR-T cell therapy (4, 10, 38, 53), one could consider vaccination strategies beginning 6–12 months after CAR-T cell therapy as recently detailed in an expert guideline (37). Vaccinations before CAR-T cell therapy, as is preferred in solid organ transplant recipients (54), is another strategy worth considering, although vaccination in the context of recent chemotherapy and refractory or relapsed malignancy may have limited immunogenicity.

To our knowledge, these are the most comprehensive data examining pathogen-specific antibodies in CAR-T cell therapy recipients and provide the first insights into pathogen-specific immunity after BCMA-CAR-T cell therapy. We evaluated clinical and immunologic variables to provide hypothesis-generating data about differential roles of CD19^+^ versus BCMA^+^ B cells in maintaining pathogen-specific antibodies. It is important to note that IgG levels above defined thresholds do not necessarily imply protection from infection or disease, and thresholds established in healthy individuals may not apply to immunocompromised individuals. A correlation between lower infection or disease rates and pathogen-specific antibody levels has been extensively characterized for many but not all vaccine-preventable infections (55). Antibody tests and thresholds used for seroprotection vary and may affect comparisons with other data. Cellular immunity and other host responses are additional factors that modulate infection risk and severity that were not considered in this study (56). Due to the challenge of performing studies with endpoints of proven infections, antibody titers are accepted correlates, and in some instances surrogates, for seroprotection in immunocompromised populations (47, 55, 57). Our sample size precluded adjustment for heterogenous participant and CAR-T cell product characteristics for the primary outcome, but all individuals had sustained remissions, indicating effective depletion of the targeted B cells. We stratified our results by receipt of IGRT within 16 weeks, as “false-positive” serologic results are unlikely beyond 16 weeks after IGRT (58, 59). Participants who received IGRT within 16 weeks were excluded from the analyses of pathogen-specific IgG outcomes and may be different from those not receiving IGRT; trials that randomize individuals to IGRT versus no IGRT are needed to better understand these findings. Inclusion of geographically diverse individuals improves the generalizability of our findings. Lower levels of pathogen-specific IgG in BCMA- compared with CD19-CAR-T cell therapy recipients may be related to a number of differences between the 2 populations, including underlying disease, prior plasma cell targeted therapies, more frequent prior HCT, longer time from HCT to CAR-T cell therapy, and shorter duration between CAR-T cell infusion and sample collection; these findings warrant replication in larger cohorts. The majority of CD19-CAR-T cell therapy recipients (77%, Supplemental Table 1) and all BCMA-CAR-T cell recipients received a product with a 4-1BB costimulatory domain. CAR-T cell persistence may differ between 4-1BB and CD28 costimulated products, and this may limit the generalizability of our findings to non–4-1BB costimulated products.

In summary, this study demonstrates that a high proportion of adults with long-term remission after CD19-CAR-T cell therapy for B cell malignancies had seroprotective IgG titers for vaccine-preventable infections. Seroprotection for certain pathogens, such as *S*. *pneumoniae*, was infrequent and indicates the need for studies of vaccination strategies. We demonstrate provocative data that BCMA-CAR-T cell therapy recipients had less pathogen-specific IgG and fewer seroprotective titers to vaccine-preventable infections. Thus, they may be at increased risk for infections and benefit most from IGRT and ultimately complete revaccination as is done after HCT. Longitudinal data are needed to better understand the direct effect of CAR-T cell therapies on pathogen-specific antibodies. Prospective studies of vaccine immunogenicity and the impact of IGRT on infection incidence in children and adults are important next steps to improve resource allocation and long-term care of these individuals.

## Methods

### Study design and participants.

We conducted a prospective cross-sectional observational study of children and adults who had a sustained remission of their underlying B lineage malignancy after they received CD19- or BCMA-CAR-T cell therapy at Fred Hutchinson Cancer Research Center (Fred Hutch) or Seattle Children’s Hospital. We screened all individuals who received commercial CD19-CAR-T cell therapy or investigational CD19- or BCMA-CAR-T cell therapy under a study protocol at Seattle Children’s Hospital (NCT02028455, NCT03244306, NCT03330691, NCT03338972, and NCT03684889) and Fred Hutch (NCT01865617, NCT02706392, NCT03103971, NCT02706405, NCT02631044, NCT03277729, NCT03105336, NCT02614066, NCT03331198, NCT03575351, NCT03502577, and NCT03338972). Individuals were eligible if they were more than 6 months from CAR-T cell therapy, were alive, were in remission, and had not received an HCT or other new antitumor treatments after CAR-T cell therapy. Use of preplanned maintenance therapies started at the time of CAR-T cell therapy was not an exclusion criterion. All individuals meeting eligibility criteria were approached as depicted in Figure 1. One individual who received vaccines against 7 pathogens after CAR-T cell therapy was excluded.

### Samples.

Blood was collected once per participant, transported to our laboratory, processed, and cryopreserved for batch testing. We isolated serum from 10–20 mL of whole blood collected in clot activator red-top vacutainers and stored at –80°C. We isolated PBMCs from 10–20 mL of whole blood collected in acid citrate dextrose vacutainers. PBMCs were isolated by layering 10 mL of blood onto 10 mL of Ficoll-Histopaque followed by centrifugation at 300*g* for 15 minutes at room temperature to obtain a mononuclear cell layer. PBMCs were removed by transfer pipet, washed twice with phosphate-buffered saline, resuspended in a mixture of 90% fetal bovine serum and 10% DMSO, aliquoted, cooled in a controlled rate freezing container at –80°C, and stored in liquid nitrogen.

### Testing for serum total immunoglobulins and vaccine-preventable infection IgG titers.

We measured serum levels of total IgG, total IgM, and total IgA at the University of Washington Department of Laboratory Medicine using turbidometry. Normal ranges by age are detailed in Supplemental Table 2. For immunoglobulin values below the lower detection limit, we assigned values of the lower detection limit divided by 2. We measured pathogen-specific IgG for 12 vaccine-preventable infections consisting of HAV, HBV, varicella zoster virus, measles (rubeola), mumps, rubella, Hib, *Clostridium tetani*, *Corynebacterium diphtheriae*, *B*. *pertussis*, *S*. *pneumoniae* (23 serotypes), and poliovirus. Testing was done by gold standard tests in Clinical Laboratory Improvement Amendments–certified reference laboratories. Details of testing and results interpretation are in Supplemental Tables 3 and 4. Equivocal results were considered negative.

### Testing for IgG to viral and bacterial epitopes using a systematic epitope scanning method (VirScan).

We used a comprehensive serological profiling assay (VirScan) to measure the diversity of a participant’s pathogen-specific IgG repertoire. VirScan uses bacteriophages to display a synthetic pathogen epitope library, immunoprecipitation to extract antibody-epitope interactions, and massively parallel sequencing to analyze DNA of antibody-bound phages (19, 60, 61). The synthetic peptides of the VirScan library span the reference protein sequences (collapsed to 90% identity) of all viruses annotated to have human tropism in the UniProt database (244 species, 119,233 epitopes) as well as full-length proteins of 62 bacterial species (2986 epitopes) identified from the Immune Epitope Database and extracted from the UniProt database (61). To create the bacteriophage library, a DNA microarray is used to synthesize 200-mer oligonucleotides. The oligonucleotides encode 56-residue peptide tiles with 28-residue overlaps and are cloned into a T7 bacteriophage display vector (60). *E*. *coli* is used for amplification. For this study, we reamplified and sequenced the VirScan 2.0 library (61) from an aliquot provided by Stephen Elledge (Harvard Medical School, Boston, Massachusetts, USA) according to the T7Select manufacturer’s protocol (Novagen, MilliporeSigma). We mixed the library with a sufficient volume of serum to provide 2 μg of IgG in each of 2 replicates per sample and incubated the mixture for 20 hours at 4°C with constant mixing, followed by immunoprecipitation of the bound antibody-phage complexes after 4 hours of continuous mixing with protein A and G magnetic beads (Invitrogen, Thermo Fisher Scientific) at 4°C. After the removal of unbound phage particles, precipitated samples were lysed to release corresponding DNA sequences from the bound phage, followed by 2 rounds of PCR, the first to amplify the phage inserts and the second to attach appropriate adapter sequences and individual index sequences to each sample to allow pooling. Indexed amplifiers from the second PCR were quantified with fluorometry using the Qubit dsDNA HS (High Sensitivity) Assay Kit (Thermo Fisher Scientific), pooled in equal proportions, and gel purified, and the readable library was quantified by KAPA qPCR (Roche). The pool was sequenced on an Illumina HiSeq 2500 (NextGen Sequencing) using a custom read primer. Primer sequences and PCR cycling conditions followed Xu et al. (60). Sequence reads were aligned, and oligonucleotides were deconvoluted into epitope hits using a maximum parsimony approach to capture the probability that sequences were enriched in a given sample over their frequency in the original library as described elsewhere (60). Laboratory work and sequencing were blinded to sample identity.

### Evaluation of B cell and T cell subsets.

We immunophenotyped B cells and T cells from PBMCs. Additionally, we immunophenotyped RSV-specific B cells in study participants and 3 healthy adult controls by using the RSV fusion glycoprotein in the prefusion conformation (preF), which is targeted by the majority of RSV-specific neutralizing antibodies in human sera (40). We thawed PBMCs quickly at 37°C and incubated them immediately for 30 minutes on ice in 100 μL of FACS buffer containing a cocktail of antibodies prior to washing and analysis on a FACSymphony (BD). FACS buffer consisted of 1× DPBS containing 1% newborn calf serum (Life Technologies, Thermo Fisher Scientific). Cells were labeled with antibodies including combinations of anti-CD4 Alexa Fluor 488 (clone OKT4, BioLegend), anti-IgM PerCP-Cy5.5 (clone G20-127, BD), anti-EGFR APC (cetuximab, clone MAB9577; R&D Systems, Bio-Techne), anti-CD8 APC-H7 (clone SK1, BD), anti-CD19 BV421 (clone HIB19, BD), anti-CD45 BV510 (clone HI30, BD), anti-CD3 BV605 (clone UCHT1, BioLegend), anti-CD14 BV711 (clone M0P-9, BD), anti-CD16 BV711 (clone 3G8, BD), anti-CD20 BUV395 (clone 2H7, BD), anti-CD38 BUV661 (clone HIT2, BD), anti-IgD BUV737 (clone IA6-2, BD), anti-CD27 PE-Cy7 (clone LG.7F9, Thermo Fisher Scientific), a fixable viability dye (FVD), a tetramer of RSV preF conjugated to PE, and a tetramer of 6x HIS-tag conjugated to PE-Dylight 650 (Barney Graham, NIH, Bethesda, Maryland, USA) (40). The tetramer of 6x HIS-tag allowed the exclusion of B cells binding to either the HIS-tag or PE. B cells were defined as CD19-expressing cells in the lymphocyte population. The following B cell populations were delineated: CD27^–^CD38^+^IgM^hi^IgD^lo^ (transitional B cells), CD27^–^CD38^–^IgD^+^ (naive B cells), CD27^+^IgD^–^ (switched memory B cells), and live RSV preF-specific B cells (FVD^–^CD14^–^CD16^–^CD3^–^CD45^+^CD19^+^HIS^–^RSV preF^+^). RSV-specific B cells were further delineated into the above-described B cell populations. In the healthy controls, we used a similar panel that did not include anti-EGFR. Absolute B and T cell counts were calculated by multiplying proportions from flow cytometry by absolute lymphocyte counts from complete blood cell count results. Analyses were performed using FlowJo Software version 10.7.1.

### Outcomes.

The primary outcome was the proportion of participants with pathogen-specific IgG levels above a threshold considered to correlate with protection from vaccine-preventable infections (Supplemental Table 3) (55). We refer to these levels as “seroprotective” in this study. An additional outcome was the total number of pathogen-specific viral and bacterial epitopes to which IgG was detected (“epitope hits”) using VirScan (60, 61).

### Statistics.

We extracted data from medical records and electronic databases. We used nonparametric tests and Spearman’s rank correlation for bivariate comparisons. To mitigate interference from IGRT, the primary outcome was analyzed among participants who had not received IGRT within 16 weeks (≥4 half-lives for circulating IgG) before sample collection as prespecified (21, 62–64). Results from participants who received vaccinations between CAR-T cell infusion and sample collection were excluded for the corresponding pathogens. We report proportions (i.e., prevalence) of participants with seroprotective titers for vaccine-preventable infections with Wilson 95% CI. We also present absolute antibody titers after a log_10_(value+1) transformation to account for right-skewed distributions and values below 1. To test for associations between prespecified and exploratory clinical and immunological variables and seroprotective antibody levels, we used GEE with a Poisson distribution, log link, and small sample bias correction to account for within-participant correlations; this method accommodates the multiple correlated pathogen-specific binary endpoints comprising the primary outcomes for each participant. We assumed a common effect among all included pathogens (65). GEE estimates are presented as prevalence ratios with 95% CI, indicating how common seroprotective antibody levels were relative to the comparator group. We present the number of epitope hits from VirScan using violin plots and tested for associations with clinical and immunological variables using linear regression. Variables with a *P* value less than 0.1 in univariate analyses were candidates for inclusion in the multivariable model. Variables were retained in the multivariable model if their inclusion modified the CAR-T cell target variable coefficient by more than 10% or if they were significantly (at a level of less than 0.05) associated with the outcome. Prespecified primary explanatory variables were CAR-T cell target, age, total IgG, prior HCT, time after CAR-T cell infusion, and absolute CD19^+^ B cell count. Analyses were performed using Stata (16.0) and R (version 3.6.2).

### Study approval.

This study was approved by the Fred Hutch Institutional Review Board; all participants provided written informed consent prior to inclusion in the study in accordance with the Declaration of Helsinki.

## Author contributions

JAH, CSW, and EMK designed the study. CSW, JAH, JM, JKC, AVH, MB, RAG, AJC, DJG, DGM, and CJT collected data. JB and JJT performed the flow cytometry analyses. LJS and TSA performed the VirScan testing. CSW, EMK, SD, JB, JJT, and JAH analyzed the data and created the figures. CSW, EMK, JB, JJT, MB, MJB, CJT, and JAH interpreted the data. CSW and JAH drafted the initial manuscript with help from EMK. All authors contributed to the writing and revision of the manuscript and approved the final version.

## Supplementary Material

Supplemental data

Trial reporting checklists

ICMJE disclosure forms

## Figures and Tables

**Table 1:**
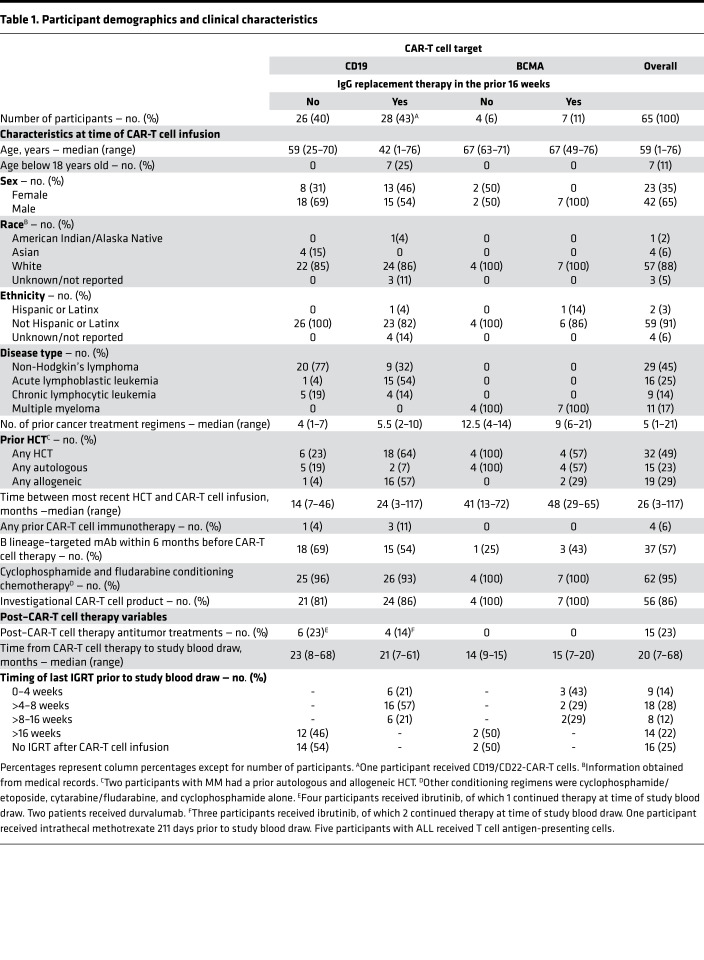
Participant demographics and clinical characteristics

## Version 1 04/29/2021

In-Press Preview

## Version 2 06/08/2021

Electronic publication
