# Supplementary material for: Antibodies against vaccine-preventable infections after CAR-T cell therapy for B cell malignancies
Source: JCI Insight. 2021 Jun 8;6(11):e146743. doi: 10.1172/jci.insight.146743 (PMC8262349; doi:10.1172/jci.insight.146743)
Supplement: Supplemental data [file jciinsight-6-146743-s142.pdf]

SUPPLEMENTARY APPENDIX TO

Antibodies to Vaccine-Preventable Infections after CAR-T-Cell Therapy for B-Cell Malignancies

TABLE OF CONTENTS

SUPPLEMENTARY FIGURES 2

Figure S1. Distribution of serum total IgG stratified by time from most recent IgG replacement therapy 2

Figure S2. B-cell subsets in 23 participants with ≥20 $CD19^+$ B-cells/µL in peripheral blood 3

Figure S3. Distribution of absolute $CD4^+$ and $CD8^+$ T-cell counts 4

Figure S4. Proportion of participants with seroprotective IgG titers to 23 *S. pneumoniae* serotypes 5

Figure S5. Absolute pathogen-specific IgG titers for *S. pneumoniae* serotypes stratified by CD19- versus BCMA-CAR-T-cell therapy 6

Figure S6. Association of secondary clinical variables with seroprotective antibody titers and epitope hits. 7

SUPPLEMENTARY TABLES 8

Table S1. Details of the CAR-T-cell products and protocols for enrolled participants 8

Table S2. Normal ranges of total IgG, IgA, and IgM 10

Table S3. Tests and interpretation of serum antibody titers to twelve vaccine-preventable infections....... 11

Table S4. Reference values for 23 *S. pneumoniae* serotypes 12

Table S5. Number of participants who were tested and had seroprotective antibody titers for each vaccine-preventable infection among 30 adults without IgG replacement therapy 13

Table S6. Association of clinical variables with epitope hits by univariate and multivariable linear regression analysis 15

CONFLICT OF INTEREST STATEMENT 17

.

SUPPLEMENTARY FIGURES

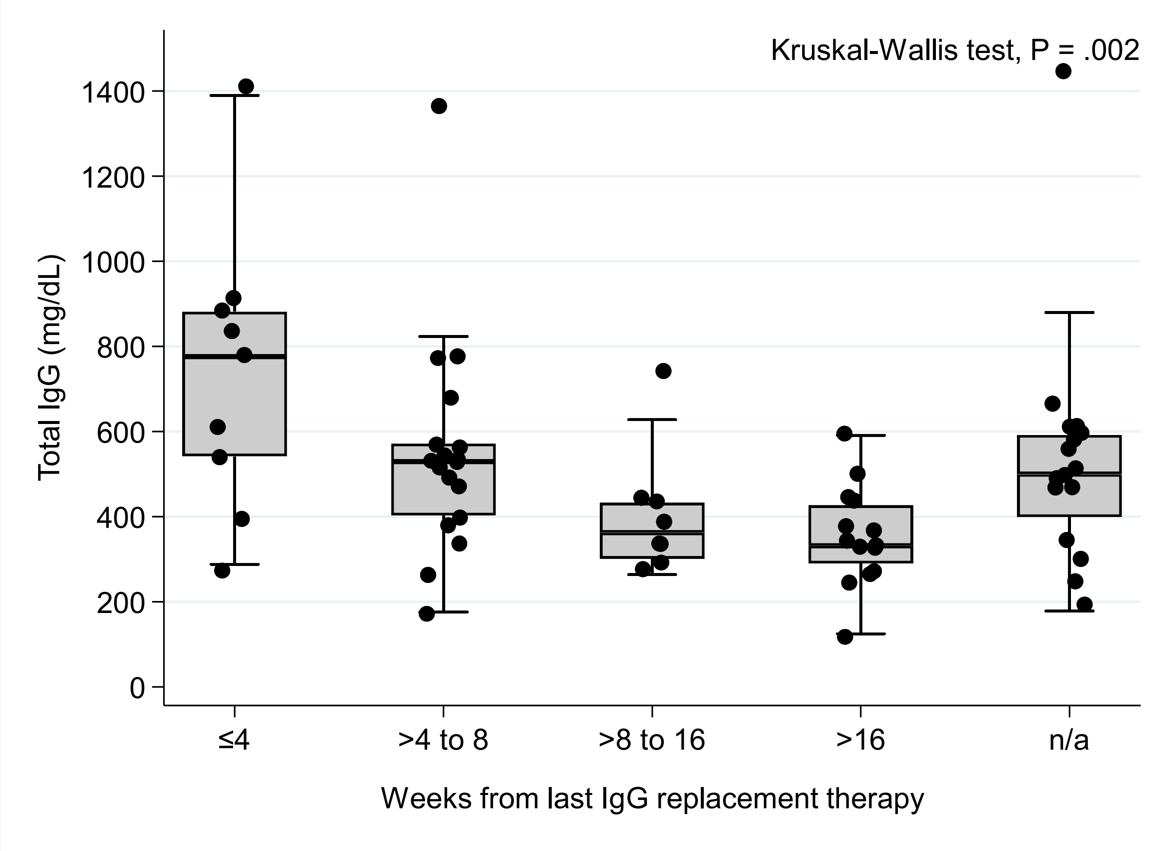

Figure S1. Distribution of serum total IgG stratified by time from most recent IgG replacement therapy (IGRT)

Each dot represents results from an individual participant (n=65). Boxes represent the interquartile range, horizontal lines represent the median, and whiskers are set at 1.5 times the interquartile range below the lower quartile and above the upper quartile. Total IgG levels differed significantly among the groups defined by timing of most recent IGRT, with higher levels among participants whose sample was obtained closer to replacement IGRT (Kruskal-Wallis test, *P* = .002). “n/a” indicates participants who did not receive IGRT between CAR-T-cell infusion and sample collection.

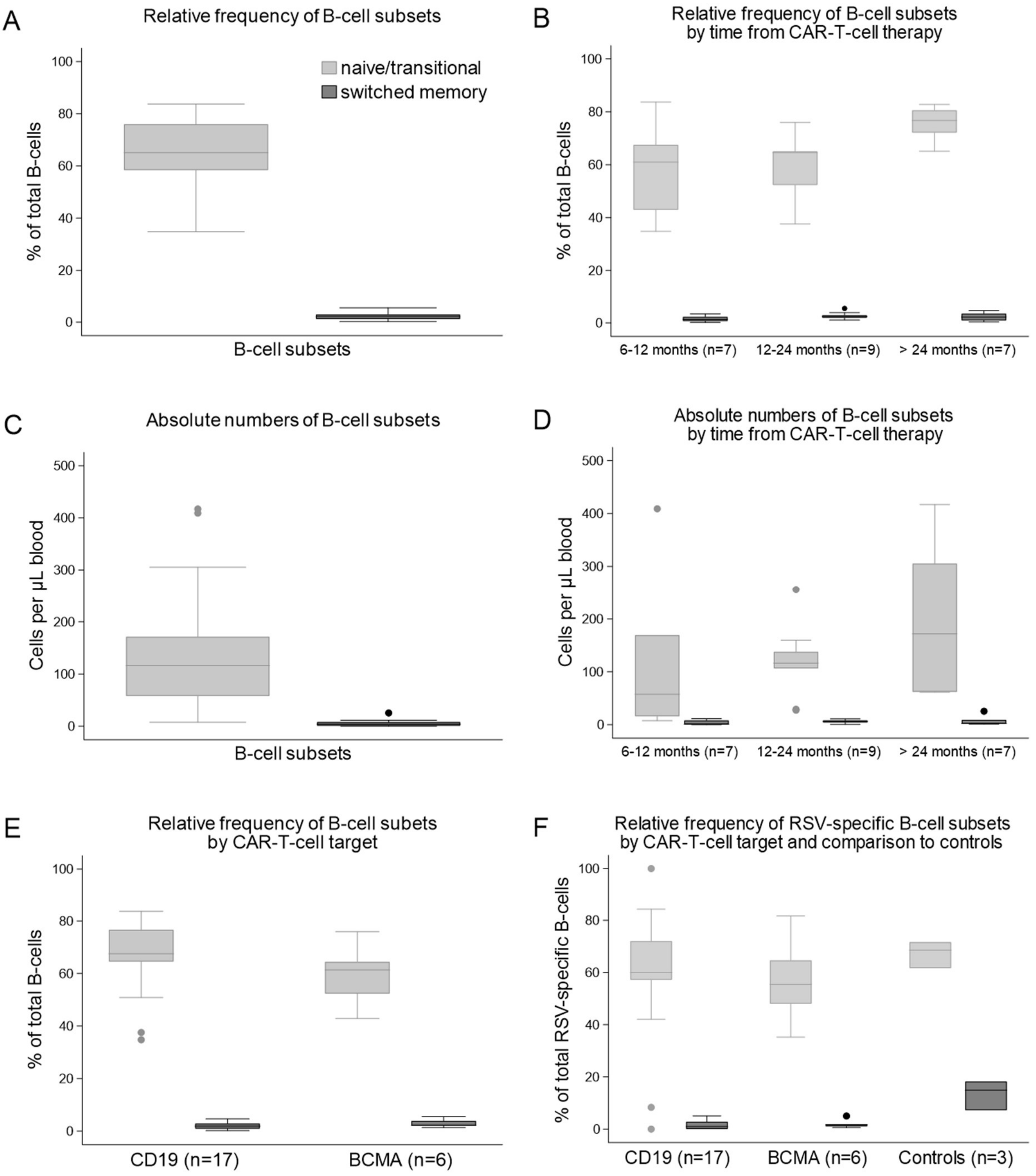

Figure S2. B-cell subsets in 23 participants with ≥20 CD19$^+$ B-cells/µL in peripheral blood
(**A**) B-cell subsets presented as percentage of total B-cells and (**C**) as absolute cell counts. (**B**) B-cell subsets presented as percentage of total B-cells stratified by time period from CAR-T-cell therapy and (**D**) as absolute cell counts stratified by time period from CAR-T-cell therapy. (**E**) B-cell subsets presented as percentages of total B-cells stratified by CAR-T-cell target. (**F**) RSV-specific B-cell subsets presented as percentage of total RSV-specific B-cells and stratified by CAR-T-cell target; also shown are data from three healthy adult controls. CD19$^+$ B-cell subsets were delineated as follow: transitional B-cells (CD27$^-$CD38$^+$IgM^{hi}IgD^{low}), naïve B-cells (CD27$^-$CD38$^-$IgD$^+$), switched memory B-cells (CD27$^+$IgD$^-$), and RSV-specific B-cells (FVD$^-$/CD14$^-$/CD16$^-$/CD3$^-$/CD45$^+$/CD19$^+$/HIS$^-$/RSV preF$^+$). In all panels, boxes represent the interquartile range, horizontal lines represent the median, whiskers are set at 1.5 times the interquartile range below the lower quartile and above the upper quartile, and dots are outliers.

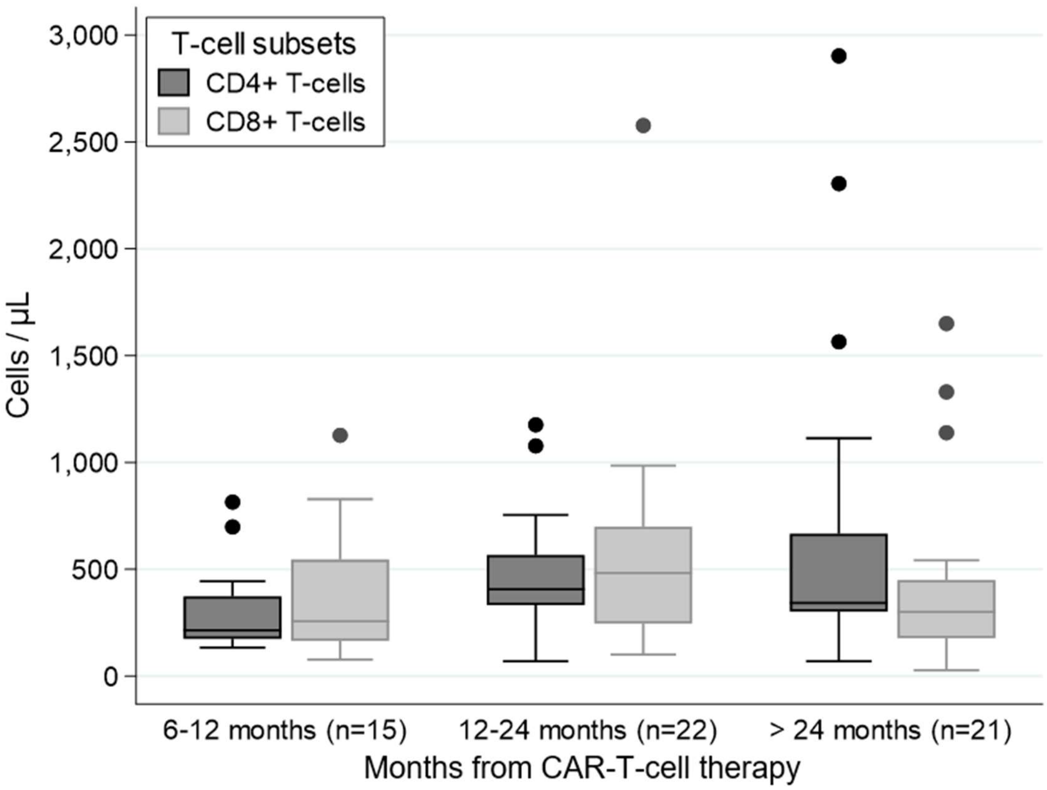

Figure S3. Distribution of absolute CD4$^+$ and CD8$^+$ T-cell counts stratified by time from CAR-T-cell therapy

These box plots illustrate the absolute CD4$^+$ and CD8$^+$ T-cell counts among 58 participants with available results, stratified by time from CAR-T-cell infusion. Boxes present the interquartile range, horizontal lines represent the median, whiskers are set at 1.5 times the interquartile range below the lower quartile and above the upper quartile, and dots are outliers. Lower limits of normal are 730 cells/µL for CD4$^+$ T-cells and 250 cells/µL for CD8$^+$ T-cells.

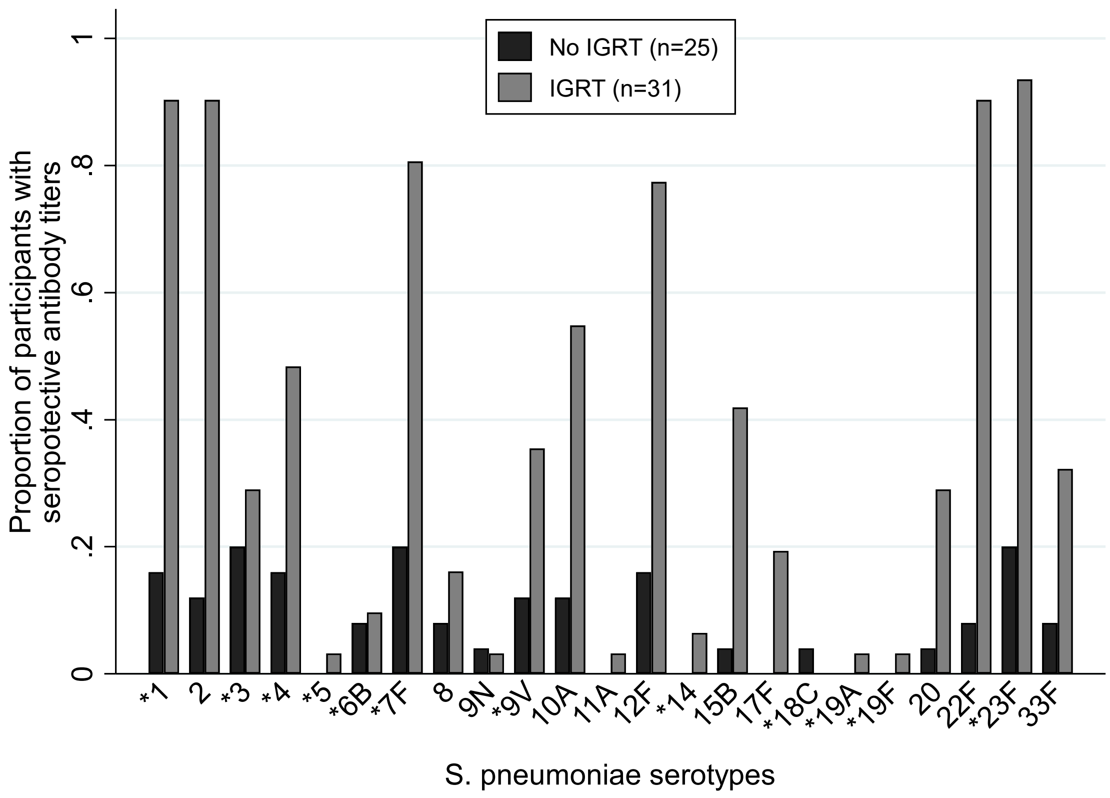

Figure S4. Proportion of participants with seroprotective IgG titers to 23 *S. pneumoniae* serotypes stratified by receipt of IgG replacement therapy (IGRT) within the previous 16 weeks

All 23 *S. pneumoniae* serotypes are contained in the 23-valent nonconjugated polysaccharide vaccines. Serotypes included in the pneumococcal conjugate vaccine (PCV13) are indicated by a *.

.

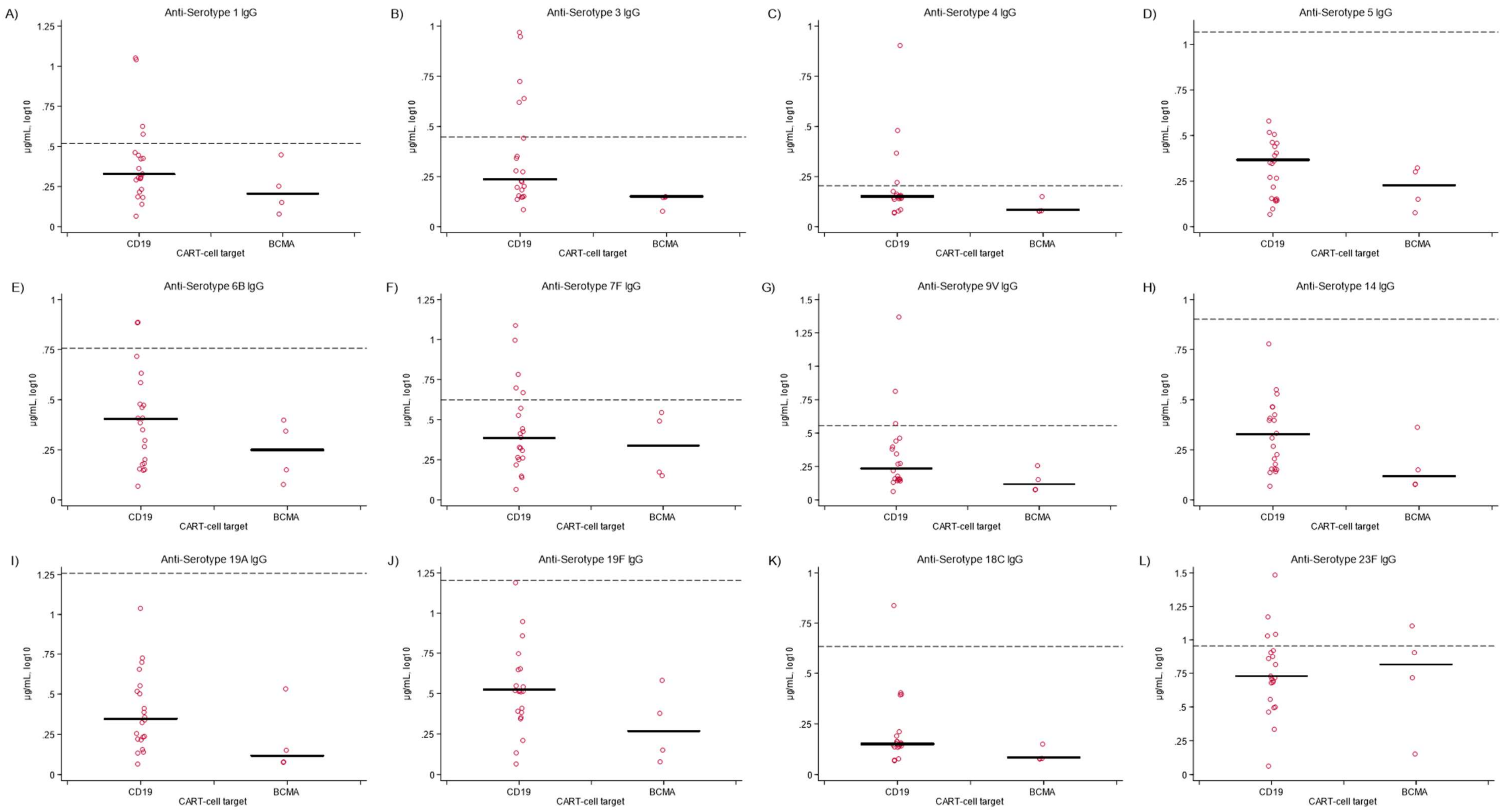

Figure S5. Absolute pathogen-specific IgG titers for *S. pneumoniae* serotypes stratified by CD19- (n=21) versus BCMA-CAR-T-cell therapy (n=4) among participants without IGRT in the previous 16 weeks

Serotypes displayed are contained in Prevnar 13. In all panels, solid black horizontal bars represent the median and horizontal dashed reference lines represent the cut-off value for seroprotection. Each data point represents a participant. Anti-IgG values were transformed using log10(value+1).

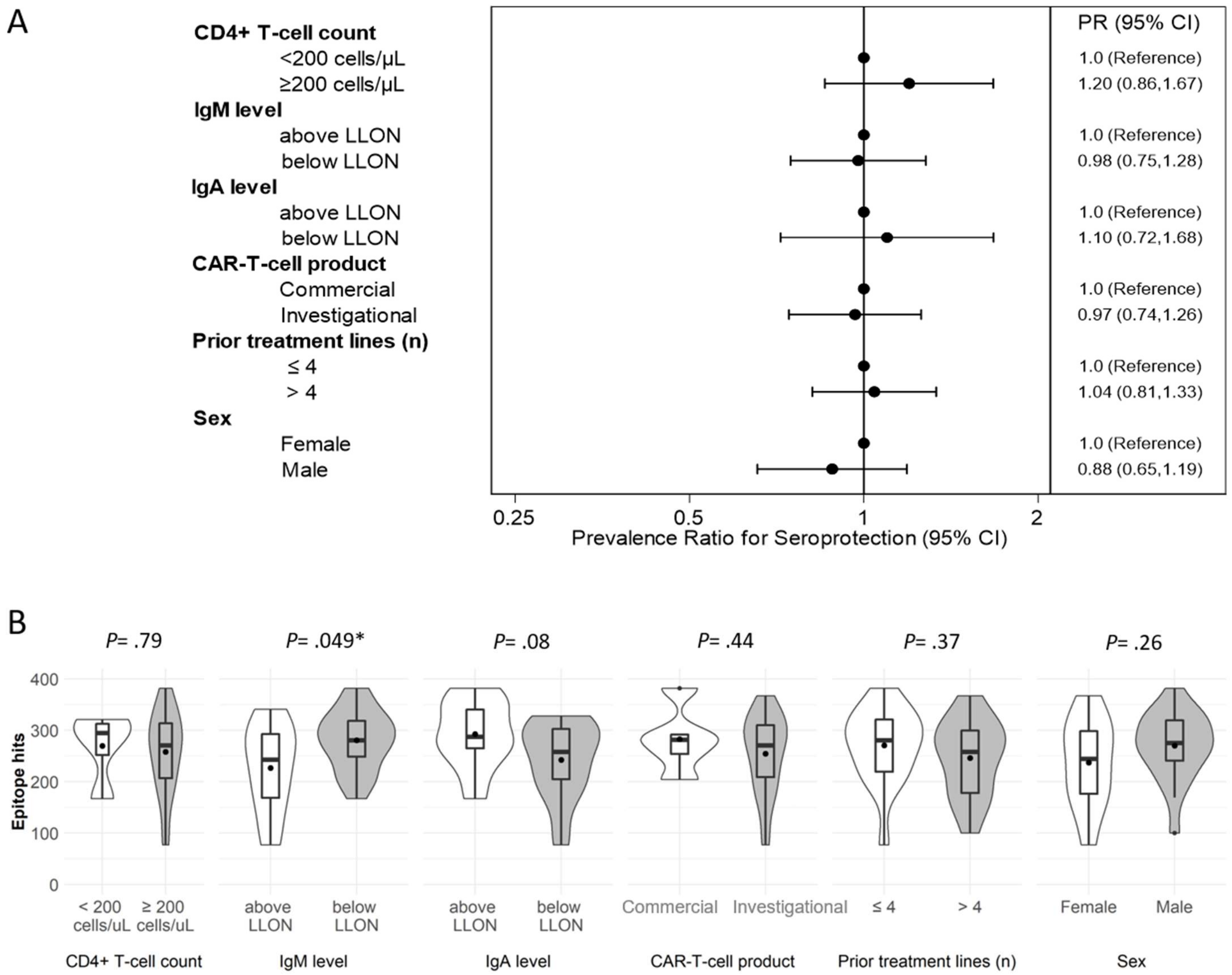

Figure S6. Association of secondary clinical variables with seroprotective antibody titers and epitope hits among 30 participants without IGRT in the previous 16 weeks

Supplementary to Figure 6 in the main manuscript, (**A**) this forest plot demonstrates associations of additional clinical variables with the prevalence of seroprotective antibody titers. Values <1 indicate a lower prevalence of seroprotective antibody titers. Dots represent prevalence ratios (PR), and whiskers indicate the 95% confidence interval (CI). LLON indicates the lower limit of normal. (**B**) Violin plots comparing the number of viral or bacterial epitopes recognized by IgG by clinical variables. Violins show the distribution of the data. Boxplots indicate the interquartile range and median. Dots in the boxes indicate the mean. P-values are derived from the univariate regression model (**Table S6**). *The IgM level variable remained significant in an adjusted model.

SUPPLEMENTARY TABLES

Table S1. Details of the CAR-T-cell products and protocols for enrolled participants

**No. of participants**	**Commercial product/study protocol**	**Study phase**	**Age**	**Underlying diseases**	**CAR-T-cell target**	**Co-stimulatory domain**
Commercial CAR-T-cell product						
9	Axicabtagene ciloleucel	n/a	adults	large B-cell lymphoma	CD19	CD28
Investigational CAR-T-cell product						
4	NCT02631044[A]	1	≥18y	NHL	CD19	4-1BB
3	NCT03105336[B]	2	≥18y	NHL	CD19	CD28
20	NCT01865617	1/2	≥18y	ALL, CLL, NHL	CD19	4-1BB
4	NCT03103971	1	≥18y	ALL, NHL	CD19	4-1BB
2	NCT02706405	1	≥18y	NHL	CD19	4-1BB
7	NCT03338972	1	>21y	MM	BCMA	4-1BB
4	NCT03502577	1	>21y	MM	BCMA	4-1BB
11	NCT02028455[C]	1/2	1-26y	ALL	CD19	4-1BB
1	NCT03330691	1	1-26y	$CD19^+$ /$CD22^+$ leukemia	CD19/22	4-1BB

ALL indicates acute lymphoblastic leukemia; CLL, chronic lymphocytic leukemia; MM, multiple myeloma; NHL, non-Hodgkin lymphoma; y, years

[A]This study used lisocabtagene maraleucel

[B]This study used axicabtagene ciloleucel

[C]Eligible individuals may have participated in protocol NCT03186118, a phase 1 pilot study for CD19 T-antigen presenting cells (T-APCs) following CAR-T-cell immunotherapy.

Table S2. Normal ranges of total IgG, IgA, and IgM by age

IgG interpretation		IgA interpretation		IgM interpretation	
**Age**	**Range (mg/dL)**	**Age**	**Range (mg/dL)**	**Age**	**Range (mg/dL)**
0-1y	327-1270	0-11m	0-83	1d-14d	5-30
2y-3y	468-1250	1y-3y	20-100	15d-5m	15-109
4y-5y	532-1340	4y-6y	27-195	6m-1y	43-239
6y-7y	454-1360	7y-9y	34-305	2y-5y	50-199
8y-9y	568-1360	10y-11y	53-204	6y-11y	50-260
10y-11y	568-1490	12y-13y	58-358	12y-16y	45-240
12y-13y	664-1490	14y-15y	47-249	≥17y	40-350
14y-17y	550-1440	16y-19y	61-348		
≥18y	610-1616	≥20y	84-499		

d indicates days; m, months; y, years.

Table S3. Tests and interpretation of serum antibody titers to twelve vaccine-preventable infections

Pathogen	Laboratory	Method	Test name, manufacturer	Threshold for positive result[a]
Hepatitis A virus (HAV)	University of Washington	Chemi-luminescence	Architect HAVAb-IgG, Abbott Diagnostics	≥1.0 signal/cutoff ratio
Hepatitis B virus (HBV), surface antibody	University of Washington	Chemi-luminescence	Architect AUSAB, Abbott Diagnostics	≥12 mIU/mL
Varicella zoster virus (VZV)	University of Washington	IFA	VZV IgG IFA, Hemagen Diagnostics	>1:8 dilution
Measles (Rubeola)	University of Washington	ELISA	ELISA Measles IgG Test System, Zeus Scientific	≥1.10 index value
Mumps	University of Washington	ELISA	ELISA Mumps IgG Test System, Zeus Scientific	≥1.10 index value
Rubella	University of Washington	ELISA	ELISA Rubella IgG Test System, Zeus Scientific	≥1.10 index value
Haemophilus influenzae type b	University of Washington	ELISA	VaccZyme *Haemophilus influenzae* type b IgG kit, Binding Site	>1.00 mg/L
Clostridium tetani	Mayo Clinic Laboratories	ELISA	Anti-Tetanus Toxoid ELISA (IgG), EuroImmun US	≥0.01 IU/mL[b]
Corynebacterium diphtheriae	Mayo Clinic Laboratories	ELISA	Anti-Diphtheria Toxoid ELISA (IgG), EuroImmun US	≥0.01 IU/mL[c]
Bordetella pertussis	Mayo Clinic Laboratories	ELISA	Anti-Bordetella pertussis toxin ELISA (IgG), EuroImmun US	≥100 IU/mL
Streptococcus pneumoniae, 23 Serotypes	Mayo Clinic Laboratories	Microsphere photometry	Streptococcus pneumoniae IgG Antibodies, 23 Serotypes, Mayo Clinic	Results ≥ reference value for at least 50% of serotypes (**Table S4**)
Poliovirus (Types 1, 3)	Quest Diagnostics	Neutralization	Poliovirus (Types 1, 3) Antibodies, Quest Diagnostics	≥1:8 dilution

[a]Positive results are referred to as seroprotective for the purposes of this study.
[b]A tetanus toxoid booster should be considered for values between 0.01 IU/mL and 0.5 IU/mL.
[c]A diphtheria toxoid booster should be considered for values between 0.01 IU/mL and <0.1 IU/mL.
ELISA indicates enzyme-linked immunosorbent assay; IFA, indirect fluorescence antibody.

Table S4. Reference values for 23 *S. pneumoniae* serotypes (Mayo Clinic Laboratories)

Serotype	Reference value (mcg/mL)	Serotype	Reference value (mcg/mL)
1 (1)	≥2.3	22F (22)	≥7.2
2 (2)	≥1.0	23F (23)	≥8.0
3 (3)	≥1.8	6B (26)	≥4.7
4 (4)	≥0.6	10A (34)	≥2.9
5 (5)	≥10.7	11A (43)	≥2.4
8 (8)	≥2.9	7F (51)	≥3.2
9N (9)	≥9.2	15B (54)	≥3.3
12F (12)	≥0.6	18C (56)	≥3.3
14 (14)	≥7.0	19A (57)	≥17.1
17F (17)	≥7.8	9V (68)	≥2.6
19F (19)	≥15.0	33F (70)	≥1.7
20 (20)	≥1.3		

Table S5. Number of participants who were tested and had seroprotective antibody titers for each vaccine-preventable infection among 30 adults without IgG replacement therapy within the previous 16 weeks

	Overall		CD19-CAR-T-cell therapy recipients		BCMA-CAR-T-cell therapy recipients	
**Vaccine-preventable infection**	No. of participants with seroprotective antibody titers / valid test results	Percentage of participants with seroprotective antibody titers (Wilson 95% CI)	No. of participants with seroprotective antibody titers / valid test results	Percentage of participants with seroprotective antibody titers (Wilson 95% CI)	No. of participants with seroprotective antibody titers / valid test results	Percentage of participants with seroprotective antibody titers (Wilson 95% CI)
Varicella zoster	24/ 26	92 (76-98)	22/ 22	100 (85-100)	2/ 4	50 (15-85)
Rubella	27/30	90 (74-97)	24/ 26	92 (76-98)	3/ 4	75 (30-95)
Polio	25/ 28	89 (73-96)	23/ 24	96 (80-99)	2/ 4	50 (15-85)
Tetanus	25/ 28	89 (73-96)	22/ 24	92 (74-98)	3/ 4	75 (30-95)
Diphtheria	25/ 28	89 (73-96)	23/ 24	96 (80-99)	2/ 2	50 (15-85)
Measles	24/ 30	80 (63-90)	24/ 25	92 (76-98)	0/ 4	0 (0-49)
Mumps	15/ 30	50 (33-67)	15/ 26	58 (39-74)	0/ 4	0 (0-49)
Hepatitis A	12/ 28	43 (27-61)	11/ 24	46 (28-65)	1/ 4	25 (5-70)
Hepatitis B	11/ 28	39 (24-58)	10/ 24	42 (24-61)	1/ 4	25 (5-70)
H. influenzae type b	4/ 27	15 (6-32)	4/ 23	17 (7-37)	0/ 4	0 (0-49)
S. pneumoniae	0/ 25	0 (0-13)	0/ 21	0 (0-15)	0/ 4	0 (0-49)
Pertussis	0/ 14	0 (0-22)	0/ 13	0 (0-23)	0/ 1	0 (0-79)

In these analyses, we included 701 of 780 pathogen-specific IgG results (90%). Seventy-nine results were not analyzed for the following reasons. Based on initial test results for pertussis demonstrating that no

patients had seroprotective antibody titers in the first batch of 31 participants, we did not test for pertussis in the second batch of the remaining 34 individuals. Four results (pertussis, diphtheria, tetanus, *S. pneumoniae*) could not be interpreted in a participant with lipemia. Forty-one results were excluded due to a corresponding vaccination after CAR-T-cell therapy and before sample collection (5 participants were vaccinated for varicella zoster, 3 for polio, 5 for tetanus/diphtheria, 1 for measles/mumps/rubella, 4 for hepatitis A, 4 or hepatitis B, 4 for *H. influenzae* type b, and 8 for *S. pneumoniae*). Equivocal results were considered as non-seroprotective and affected 1 result for varicella zoster, 2 for mumps, and 30 for *H. influenzae* type B.

Table S6. Association of clinical variables with epitope hits by univariate and multivariable linear regression analysis among 30 participants without IgG replacement therapy in the previous 16 weeks

Variables	Unadjusted β-coefficient (number of epitopes) (95% CI)	*P* value	Adjusted β-coefficient (number of epitopes) (95% CI)	*P* value
**CAR-T-cell target**				
CD19	Ref	-	-	
BCMA	-140.6 (-200.4, -80.8)	<.001	-89.6 (-157.4, -21.9)	.02
**Age**				
≤60 years	Ref	-		
>60 years	-8. 7 (-62.5, 45.2)	.75		
**Total IgG**				
≥400 mg/dL	Ref	-		
<400 mg/dL	-38.6 (-90.7, 13.6)	.16		
**Prior HCT**				
No	Ref	-	Ref	
Yes	-77.5 (-127.0, -28.0)	.005	-54.4 (-107.0, -1.8)	.05
**Time from CAR-T cell infusion**				
>1 year	Ref	-		
≤1 year vs.	-12.6 (-91.7, 66.6)	.76		
**$CD19^+$ B-cell count**				
>20 cells/μL	Ref	-	Ref	
≤20 cells/μL	61.9 (7.3, 116.6)	.04	15.3 (-30.7, 61.2)	.52
**$CD4^+$ T-cell count**				
<200 cells/μL	Ref	-		
≥200 cells/μL	-11.3 (-94.8, 72.2)	.79		

**IgM level**				
above LLON	Ref	-	Ref	
below LLON	54.0 (2.7, 105.2)	.049	54.3 (7.9, 100.7)	.03
**IgA level**				
above LLON	Ref	-		
below LLON	-50.7 (-104.7, 3.4)	.08		
**CAR-T cell product**				
Commercial	Ref	-		
Investigational	-28.4 (-100.0, 43.2)	.44		
**Prior treatment lines**				
≤4	Ref	-		
>4	-24.6 (-77.9, 28.6)	.37		
**Sex**				
Female	Ref	-		
Male	32.8 (-23.1, 88.6)	.26		

HCT, hematopoietic cell transplant; LLON, lower limit of normal

Conflict of interest

JJT received research funding from Vir. AVH received consulting fees from Celgene, a Bristol Myers Squibb (BMS) company. RAG served on an advisory board for Novartis; serves on a steering committee for BMS; and has a patent (US201361898387P) licensed to BMS. AJC received research funding from Janssen, Sanofi, BMS, Harpoon, and Nektar and received consulting fees from Janssen, Cellectar, Sanofi, and AbbVie. DJG has received research funding from, has served as an advisor for, and has received royalties from Juno Therapeutics, a BMS company; has served as an advisor and received research funding from Seattle Genetics and Janssen; has served as an advisor to GlaxoSmithKline, Celgene/BMS, and Legend Biotech; and has received research funding from SpringWorks Therapeutics, Sanofi, and Cellectar Biosciences. MJB received research funding from Merck, Gilead, GlaxoSmithKline, Vir, and Janssen; received consulting fees from Merck, Gilead, Astellas, Janssen, Regeneron, Moderna, Helocyte, and AlloVir; and has stock options for Helocyte. DGM has served as a consultant for A2 Biotherapeutics, Bioline Rx, Juno Therapeutics/BMS, Celgene/BMS, Kite Pharma, Gilead, Novartis, and Pharmacyclics; has received research funding, including salary support from Kite Pharma, Juno Therapeutics/BMS, and Celgene/BMS; has patents with Juno Therapeutics/BMS (pending, not issued, licensed, no royalties, no licenses); and has stock options for A2 Biotherapeutics. CJT received research funding from Juno Therapeutics/BMS, Nektar, AstraZeneca, and TCR2 Therapeutics; is a member of scientific advisory boards for Precision Biosciences, Eureka Therapeutics, Caribou Biosciences, T-CURX, Myeloid Therapeutics, ArsenalBio, and Century Therapeutics; has served on ad hoc advisory boards for Nektar, Allogene Therapeutics, PACT Pharma, AstraZeneca, Amgen, and AsherBio; has stock options for Precision Biosciences, Eureka Therapeutics, Caribou Biosciences, Myeloid Therapeutics, and ArsenalBio; and has patents pending or licensed (not issued) to Juno Therapeutics/BMS. JAH received consulting fees from Allogene Therapeutics and CRISPR Therapeutics and consulting fees and research funding from Gilead.
